# Supplementary material for: Chromothripsis during telomere crisis is independent of NHEJ, and consistent with a replicative origin
Source: Genome Res. 2019 May;29(5):737–49. doi: 10.1101/gr.240705.118 (PMC6499312; doi:10.1101/gr.240705.118)
Supplement: Supplemental Material [file supp_gr.240705.118_Supplemental_file_1.zip › contigs/annotated_contigs/DB75/contig.3.DB75_length_579_mean_cov_5.9585492228.docx]

**DB75_length_579_mean_cov_5.9585492228**

```
CCATAATCCTCACATGTCAAGGGGGAAGCCAGGTGGAGGTAATTAAATCATGGAGGCAGTTTCCCCATGTGTTCTCATGATAGTGAGTG
 >chr11:25822243-25822515 + E=5e-152 p=0e+00
AGTTCTCATGAGATCTGATGGTTTTATAAATGTGTGGTAGTTCCTCCTGTGTTCTTTCTCCTTCCCGCCATCTTGTGAAGAAGGTCCCT

TGCTTCCCCTTCACCCTCTGCTATGATCGTAAGTTTTCTGAGGCCTCCCCAGACATGCTAAACTGTGACTCAATTATACCTCTTTCATT

TATAA|TTT|ATAATTTCATTTATAAATTACCCAG|TCT|GTACTTTTTTTAAATTTTTGAGTGTTAAAAAAATACATGATGTCTTAAA
         >chrX:12448154-12448182 + E=4e-02 >chr11:25835185-25835464 + E=3e-156
ACAATAAGATTTTACAATGAAGTAAGGACTTAATATGAATAACTCTGGGTCCGTACCACATCCTCAATCCTTTGCATCATATTCATAGA

AAAATAAACAAATACATGCTAGCTTTGCTGTCAGTTTTCTTAGAGTGTTTTTATAATTTTGATATTTTAATAACAATACAAATGTATCA

TCATATTTATATAAAGAAAAATTCACATCAGAAATATGTTGGGAACACT
```
